# World Prehabilitation Day: advancing surgical optimisation beyond proof of concept

**DOI:** 10.1093/bjs/znag119

**Published:** 2026-09-16

**Authors:** Gabriele Baldini, Francesco Carli, Linda Denehy, Chelsia Gillis, Sandy Jack, Denny Levett, Daniel I McIsaac, Daniel Santa Mina, Tom Parkington, Raquel Sebio, Gerrit Slooter, Daniel Steffens, Malcolm A West

**Affiliations:** Dipartimento di Anestesia e Rianimazione, AziendaOspedaliero-Universitaria Careggi, Florence, Italy; Department of Anaesthesia, Montreal General Hospital, McGill University Health Centre, Montreal, Quebec, Canada; Centre for Health Services Research in Cancer, Peter MacCallum Cancer Centre, Melbourne, Victoria, Australia; School of Human Nutrition, McGill University, Montreal, Quebec, Canada; Anaesthesia and Critical Care Research Area, NIHR Biomedical Research Centre, University Hospital Southampton NHS Foundation Trust, Southampton, UK; Anaesthesia and Critical Care Research Area, NIHR Biomedical Research Centre, University Hospital Southampton NHS Foundation Trust, Southampton, UK; Department of Anesthesiology and Pain Medicine, University of Ottawa, Ottawa, Ontario, Canada; Faculty of Kinesiology and Physical Education, University of Toronto, Toronto, Ontario, Canada; Physical Activity, Wellbeing and Public Health Research Group, Sheffield Hallam University, Sheffield, UK; Fundació Clínic de Recerca Biomèdica–Institut D’Investigacions Biomèdiques August Pi I Sunyer (IDIBAPS), Barcelona, Spain; Department of Surgery, Máxima Medical Centre, Veldhoven, The Netherlands; Sydney Clinical Trials Centre, Faculty of Medicine and Health, The University of Sydney, Sydney, New South Wales, Australia; Anaesthesia and Critical Care Research Area, NIHR Biomedical Research Centre, University Hospital Southampton NHS Foundation Trust, Southampton, UK; Cancer Sciences, Faculty of Medicine, University of Southampton, Southampton, UK

Major surgery is one of the greatest physiological stressors encountered in healthcare. Despite advances in operative technique, anaesthesia, perioperative medicine, and Enhanced Recovery After Surgery (ERAS) pathways, postoperative complications, prolonged recovery, and loss of functional independence remain common, particularly among patients living with frailty, multimorbidity, malnutrition, or reduced physical fitness. The 16 September 2026 marks the inaugural World Prehabilitation Day and provides an opportunity to reflect on how perioperative care has evolved and to consider how prehabilitation research and clinical implementation to date have shaped modern surgical practices.

Over the past two decades, perioperative risk prediction and prehabilitation have shifted from attractive concepts to clinically relevant perioperative strategies. The biological rationale is compelling: patients enter a surgical pathway with varying levels of physiological fitness, and poor fitness has a firm relationship to poor postoperative outcomes^1^. Poor fitness may reflect a lifetime of inactivity and an age-related decline in physiological reserve, but it may also arise from diseases that reduce physical fitness or iatrogenic consequences of medical therapies—for example cancer cachexia and treatment^2,3^. Traditional perioperative pathways have often treated perioperative risk as a fixed, unmodifiable quantity. It is now recognized that impaired fitness, malnutrition, and psychological distress are measurable perioperative risk factors and, importantly, that each represents a modifiable target for intervention before surgery^4,5^. Modern perioperative pathways have been re-engineered to include early screening, risk assessment, and prehabilitation interventions. These reframe the interval between diagnosis and surgery as a therapeutic window of opportunity to improve fitness, nutritional status, and psychological well-being, resulting in improved patient resilience before a surgical insult, improving postoperative and longer-term outcomes^6^.

The evidence supporting this approach has matured substantially. Early studies in gastrointestinal cancer surgery demonstrated that neoadjuvant cancer treatment impaired physical fitness before surgery and that lower preoperative fitness is associated with poorer surgical outcomes^1,2^. These findings highlight the importance of intervening before surgery rather than focusing exclusively on postoperative recovery. Unimodal, quasi-randomized prehabilitation studies in the UK and Canada subsequently developed into larger multimodal, international RCTs of tailored exercise, nutrition, and psychological support, which have strengthened the evidence base^7,8^. These include the work by Barberan-Garcia *et al*.^9^, which demonstrated a reduction in postoperative complications among high-risk patients undergoing major abdominal surgery, while the international PREHAB trial^10^ subsequently reported reductions in severe postoperative complications together with improved postoperative functional capacity after colorectal cancer resection. Collectively, these studies established prehabilitation as an intervention capable of influencing outcomes that mattered to surgeons and patients alike. More recently, the PREPARE trial^11^ tested home-based prehabilitation in older adults living with frailty across 13 Canadian centres. This found no reduction in 30-day disability or complications; however, patients who achieved >75% adherence to the prescribed programme had significantly lower disability scores. This highlights the importance of including behaviour change strategies aimed at improving adherence to prescribed interventions^6^. A recent network meta-analysis incorporating 186 randomized trials and >15 000 patients concluded that exercise-based and multimodal prehabilitation interventions were among the most effective approaches for improving postoperative outcomes^5^.

Heterogeneity in intervention design and outcome reporting, however, remains considerable. As the field matures, questions have shifted from whether prehabilitation works to understanding optimal intervention dose, adherence, fidelity of delivery, and the biological mechanisms responsible for clinical benefit. The findings of the PREPARE trial^11^ and the development of the SOS-Prehab^12,13^ reporting standards highlight the urgent research need to achieve a common language and to translate efficacy into real-world effectiveness. Consistent language and trial reporting are imperative for interventional comparisons. The SOS-Prehab guideline is a significant step towards ensuring interventions and adherence are described with the rigour needed for meaningful comparison across trials, with a complementary core outcome set for prehabilitation research now under development^12,13^. Collectively, the central message is consistent: surgical risk is modifiable and targeted prehabilitation interventions can improve recovery from major surgery.

The next challenge is implementation rather than efficacy. Despite the growing evidence base, international survey data demonstrate that routine prehabilitation remains scantly available^14^. Moreover, relatively few centres employ standardized approaches to identify patients most likely to benefit. As healthcare systems face increasing resource pressures, universal provision of intensive prehabilitation may be neither practical nor necessary. Consequently, interest has shifted towards perioperative screening and assessment as core components of the perioperative medicine pathway. The future of prehabilitation is likely to lie in precision rather than uniform prehabilitation delivery. Risk assessment should extend beyond estimating perioperative mortality alone and focus on identifying modifiable deficits in physiological reserve, physical fitness, nutrition state, frailty, and psychological well-being. Such an approach enables targeted intervention, directing finite resources towards patients with the greatest capacity to benefit while avoiding unnecessary intervention in lower-risk individuals. Screening and assessment tools must therefore be refined, validated, and embedded within routine surgical pathways to serve as a springboard for personalized prehabilitation prescription. These principles are reflected in the 2025 *Prehabilitation for People with Cancer: Clinical and Implementation Guidelines*^6^.

Importantly, prehabilitation should not be viewed as a standalone intervention. Rather, it represents a component of the perioperative pathway continuum. Identification of perioperative risk, mitigation of modifiable factors, prehabilitation before surgery, ERAS, and rehabilitation after surgery share a common objective: improving functional recovery and patient-centred outcomes. Integrating these components within a single perioperative medicine framework offers a more coherent approach than maintaining separate services operating across different stages of the surgical journey^15,16^.

Increasing pressures to improve outcomes, reduce complications, and manage healthcare costs have further focused attentions on interventions that enhance patient resilience across the whole of the perioperative patient pathway. Emerging health-economic evaluations suggest that prehabilitation may generate value through reductions in postoperative morbidity, length of stay, critical care utilization, and readmission, supporting the case for investment at a system level^17–19^. However, widespread implementation remains limited by workforce constraints, fragmented funding models, and the absence of standardized pathways for identifying patients most likely to benefit. In the UK’s National Health Service (NHS), the new National Clinical Audit of Perioperative Care (NCAPC)^20^ represents an important step towards addressing these challenges. Designed to evaluate care across the entire elective perioperative pathway, NCAPC aims to reduce unwarranted variation in practice, promote evidence-based perioperative care, and support quality improvement initiatives.

World Prehabilitation Day therefore arrives at an important moment for surgery and the international community. The debate is no longer whether prehabilitation works, but how best to identify the patients who will benefit most and to integrate programmes into routine perioperative care and deliver them efficiently at scale. Future research should prioritize risk-stratified interventions, implementation and behaviour science, health-economic evaluation, biological mechanisms, and pragmatic clinical trials embedded within routine surgical pathways.

For surgeons, the message is clear. Prehabilitation has moved beyond proof of concept. The next decade should focus on developing healthcare policy where prehabilitation is embedded within an integrated perioperative medicine model in which screening, risk assessment, optimization, surgery, enhanced recovery, and rehabilitation are seen not as separate episodes of care, but as components of a continuous pathway designed to improve outcomes for patients.
